# 193 An exploratory review of the sedentary behaviours of clinical populations in Northern Ireland

**DOI:** 10.1093/eurpub/ckae114.130

**Published:** 2024-09-26

**Authors:** Annette Henderson, Judy Bradley, Jill Costley, Jennifer Jones, Orlagh O’Shea, Jason Wilson, Brenda O’Neill

**Affiliations:** Ulster University, Derry, United Kingdom; Wellcome-Wolfson Institute of Experimental Medicine, Belfast, United Kingdom; Ulster University, Derry, United Kingdom; University of Galway, Galway, Ireland; Royal College of Surgeons in Ireland University of Medicine and Health Sciences, Dublin, Ireland; Ulster University, Derry, United Kingdom; Ulster University, Derry, United Kingdom

## Abstract

**Purpose:**

To describe the sedentary behaviours of cardiorespiratory clinical populations in Northern Ireland. Increasing evidence highlights adults should limit their sedentary time to less than 9.5 hours/day as such behaviour increases cardiovascular and all-cause mortality. Sedentary behaviours refer to activities typically sitting or lying that do not substantially increase energy expenditure above resting.

**Development:**

UK physical activity guidelines recommend that adults should aim to be physically active daily and minimise sedentary time by breaking up periods of inactivity. Implementation of these recommendations is challenging unless measurement of sedentary behaviours in clinical settings in Northern Ireland is feasible.

**Implementation:**

Three studies quantified sedentary behaviours (SB) in cardiorespiratory populations in Northern Ireland. 63 people with bronchiectasis wore an ActiGraph; 53 had valid data with SB duration of 10 hours 34 mins (± 77 mins) (Bradley et al. 2015). 50 participants with COPD also wore an ActiGraph; 45 had valid data and SB duration of 10 hours and 45 minutes (± 81 mins) (O’Shea et al. 2015; O’Neill et al. 2018); 105 people joining a cardiac rehabilitation program wore an activPAL; 101 had valid data and SB of 10 hours 32 mins (± 2 hours 14 mins) and on program completion SB of 9 hours 44 mins (± 2 hours 33 mins) (Jones et al. 2022).

**Evaluation:**

Measuring the postural aspect of sedentary behaviour using an accelerometer (ActiGraph or activPAL), appears acceptable and feasible in clinical settings (91% valid data). SB in all 3 studies exceeded the recommended 9.5 hours/day. However, SB is modifiable and inclusion of objective measurement of SB in clinical practice would enable targets to be set to support patients to change.

**Dissemination:**

Evaluating interventions that address high levels of SB in cardiorespiratory populations could inform clinical practice guidelines. Determining the most effective device and protocol to evaluate SB, would also enable SB evaluation to become part of routine chronic disease management plans.

**Conclusion:**

Sedentary behaviours in clinical populations demonstrate patients currently exceed recommendations. Raising awareness, as well as further research testing interventions to reduce SB could drive changes in policy and practice to improve outcomes for these patients.

